# AMPA-ergic regulation of amyloid-β levels in an Alzheimer’s disease mouse model

**DOI:** 10.1186/s13024-018-0256-6

**Published:** 2018-05-15

**Authors:** Jane C. Hettinger, Hyo Lee, Guojun Bu, David M. Holtzman, John R. Cirrito

**Affiliations:** 1 Department of Neurology, Knight Alzheimer’s Disease Research Center, Hope Center for Neurological Disorders, Washington University School of Medicine, Campus Box 8111, 660 South Euclid Avenue, St. Louis, MO 63110 USA; 2 Department of Neuroscience, Mayo Clinic, Jacksonville, FL 32224 USA

**Keywords:** Alzheimer’s disease, Amyloid-beta, AMPA, Clearance, IL-6, Microdialysis

## Abstract

**Background:**

Extracellular aggregation of the amyloid-β (Aβ) peptide into toxic multimers is a key event in Alzheimer’s disease (AD) pathogenesis. Aβ aggregation is concentration-dependent, with higher concentrations of Aβ much more likely to form toxic species. The processes that regulate extracellular levels of Aβ therefore stand to directly affect AD pathology onset. Studies from our lab and others have demonstrated that synaptic activity is a critical regulator of Aβ production through both presynaptic and postsynaptic mechanisms. AMPA receptors (AMPA-Rs), as the most abundant ionotropic glutamate receptors, have the potential to greatly impact Aβ levels.

**Methods:**

In order to study the role of AMPA-Rs in Aβ regulation, we used in vivo microdialysis in an APP/PS1 mouse model to simultaneously deliver AMPA and other treatments while collecting Aβ from the interstitial fluid (ISF). Changes in Aβ production and clearance along with inflammation were assessed using biochemical approaches. IL-6 deficient mice were utilized to test the role of IL-6 signaling in AMPA-R-mediated regulation of Aβ levels.

**Results:**

We found that AMPA-R activation decreases in ISF Aβ levels in a dose-dependent manner. Moreover, the effect of AMPA treatment involves three distinct pathways. Steady-state activity of AMPA-Rs normally promotes higher ISF Aβ. Evoked AMPA-R activity, however, decreases Aβ levels by both stimulating glutamatergic transmission and activating downstream NMDA receptor (NMDA-R) signaling and, with extended AMPA treatment, acting independently of NMDA-Rs. Surprisingly, we found this latter, direct AMPA pathway of Aβ regulation increases Aβ clearance, while Aβ production appears to be largely unaffected. Furthermore, the AMPA-dependent decrease is not observed in IL-6 deficient mice, indicating a role for IL-6 signaling in AMPA-R-mediated Aβ clearance.

**Conclusion:**

Though basal levels of AMPA-R activity promote higher levels of ISF Aβ, evoked AMPA-R signaling decreases Aβ through both NMDA-R-dependent and -independent pathways. We find that evoked AMPA-R signaling increases clearance of extracellular Aβ, at least in part through enhanced IL-6 signaling. These data emphasize that Aβ regulation by synaptic activity involves a number of independent pathways that together determine extracellular Aβ levels. Understanding how these pathways maintain Aβ levels prior to AD pathology may provide insights into disease pathogenesis.

## Background

Alzheimer’s disease (AD) follows a protracted course with pathology detected years, even decades before clinical symptoms manifest. The preclinical stage of AD appears to be initiated by the aggregation of the peptide amyloid-β (Aβ) into toxic oligomers and plaques within the brain extracellular space, thereby triggering a host of biochemical and cellular pathological events [1–3]. The shift from normal production of soluble Aβ to its pathogenic aggregation is heavily influenced by the concentration of Aβ. Consequently, the rate at which Aβ is produced and secreted from the neuron, as well as its clearance from the extracellular space, appears to be directly linked to the formation of toxic amyloid species [4–6].

Our lab and others have shown that an important regulator of extracellular Aβ levels is synaptic activity [7, 8]. Elevated synaptic activity drives clathrin-mediated endocytosis at the presynaptic membrane, thereby increasing endocytosis of the amyloid precursor protein (APP) and subsequent Aβ generation [9]. At the systems level, the regional distribution of amyloid plaque deposition in AD brains correlates with default mode network connectivity, suggesting that chronic high levels of network activity contribute to plaque formation [10, 11]. However, not all increased neuronal activity results in increased Aβ concentrations. Indeed, a number of postsynaptic receptors have been shown to decrease Aβ production. Stimulation of serotonin receptors activates the extracellular regulated kinase (ERK) signaling pathway, which enhances α-secretase activity and non-amyloidogenic APP processing [12, 13]. NMDA receptor (NMDA-R) activation regulates Aβ levels bidirectionally – low concentrations of NMDA elevate Aβ levels through increased presynaptic membrane endocytosis, while higher concentrations of NMDA decrease Aβ production through dendritic, calcium-dependent signaling and increased α-secretase activity [12]. These experiments show that the relationship between neuronal activity and Aβ production is complex, with even the same receptors in some cases having opposing effects depending on the extent of activation.

AMPA receptors (AMPA-Rs) are the predominant postsynaptic glutamate-gated ion channels and are responsible for the majority of fast excitatory transmission in the CNS, making them well positioned to impact the relationship between Aβ levels and synaptic activity. Furthermore, growing evidence suggests AMPA-Rs can act as independent activators of second messenger signaling in addition to their well-established role as the primary agents of postsynaptic depolarization [14–18]. Most of the research involving AMPA-Rs and AD has focused on the deleterious effect of pathological amyloid species on AMPA-Rs [19–21], while the inverse relationship, that of AMPA-R’s effects on Aβ, has received much less attention. A notable exception is a compelling study by Hoey and colleagues, which reported increased non-amyloidogenic processing of APP following calcium-permeable AMPA-R activation in primary cortical neurons [22]. Given the AMPA-R’s dominant role in synaptic transmission and its active signaling capabilities, we hypothesized that AMPA-Rs regulate Aβ metabolism.

Using in vivo microdialysis, we found that baseline AMPA-R activity maintains higher levels of Aβ, whereas evoked activation of AMPA-Rs leads to reduced Aβ levels in the interstitial fluid (ISF) of the mouse hippocampus. Interestingly, the effect of exogenous AMPA treatment resolves into two phases. Initially, AMPA-Rs decrease Aβ levels through synaptic release of glutamate and downstream activation of NMDA-Rs. After prolonged treatment with AMPA, however, Aβ levels are reduced through an NMDA-R-independent pathway that does not rely on presynaptic transmission. Surprisingly, we found that AMPA-Rs directly influence Aβ levels by altering Aβ clearance, implicating synaptic activity with clearance mechanisms. Moreover, data collected from IL-6 deficient mice indicate a critical role for IL-6 signaling in this pathway. These findings highlight the complexity behind the overlapping pathways regulating extracellular Aβ levels.

## Methods

### Animals

The mice used for these studies were hemizygous *APPswe/PS1ΔE9* (APP/PS1) and bred on a wild-type C3H/B6 background, C57BL/6j-IL-6^*tm1Kopf*^ mice (hereafter referred to as IL-6^−/−^ mice), or littermate controls (WT) [23, 24].

Original APP/PS1 transgenic breeders as well as IL-6^−/−^ mice were purchased from Jackson Laboratory (Bar Harbor, Maine), and colonies were maintained at Washington University. Equal numbers of male and female mice were used in each study at 2–4 months of age. All studies were performed in accordance with the guidelines of AAALAC and the IACUC at Washington University.

### Aβ microdialysis

In vivo microdialysis was performed in awake and behaving APP/PS1 mice as previously described [12, 25]. Briefly, guide cannulas (BR-style, Bioanalytical Systems, West Lafayette, IN) were stereotaxically implanted above the left hippocampus, coordinates bregma − 3.1 mm, 2.5 mm lateral to midline, and 1.2 mm below dura at a 12° angle. The cannulas were securely affixed to the head with dental cement, and microdialysis probes (BR-2, 2 mm, 38 kDa MWCO, Bioanalytical Systems) were inserted into the hippocampus through the guide cannula. In APP/PS1 mice, probes were perfused with artificial cerebrospinal fluid (aCSF; 1.3 mM CaCl_2_, 1.2 mM MgSO_4_, 3 mM KCl, 04 mM KH_2_PO_4_, 25 mM NaHCO_3_, and 122 mM NaCl, pH 7.35) with 0.15% bovine serum albumin (BSA; Sigma-Aldrich, St. Louis, MO) at a rate of 1.0 μL/min with samples of hippocampal ISF collected every 90 min during basal collection or every 60 min during treatment. Because WT murine Aβ concentrations are lower than in amyloidogenic transgenic mice, microdialysis was run at 0.5 μL/min and samples collected every 3 h to increase concentration of each sample. Murine Aβ was also analyzed in the experiment using IL-6^−/−^ mice. For this experiment, microdialysis was run at 1.0 μL/min and samples were collected every 2.5 h. Basal sampling began at least 16 h following surgery. These experiments took place under constant light conditions to diminish circadian-related fluctuation in Aβ levels. At the conclusion of the experiment, all ISF samples were analyzed for either human or murine Aβ_x-40_ or Aβ_x-42_ levels by sandwich ELISA.

### Compounds

Reverse microdialysis was used to administer compounds directly into the hippocampus. Drugs were diluted into the perfusion buffer of artificial CSF and 0.15% BSA, allowing the drugs to diffuse into the brain continuously for the duration of the experiment at the same time that Aβ is collected. Due to the complexity of determining the final concentration of compound delivered to the brain, only the starting concentrations of drugs in the perfusion buffer are given. We estimate approximately 10% of the drug is delivered across the probe membrane where it is further diluted in the brain CSF. AMPA (0.5, 2, 5, 7.5, and 10 μM), MK801 (100 μM), NMDA (40 μM), and thiorphan (10 μM) were purchased from Sigma. Cyclothiazide (CTZ; 300 μM), tetrodotoxin (TTX; 5 μM), NBQX (100 μM), and GM6001 (25 μM) were purchased from Tocris Bioscience (Ellisville, MO). LY411575 (Sigma-Aldrich) was diluted in corn oil and administered subcutaneously at 5 mg/kg.

### Aβ sandwich ELISAs

ISF samples were analyzed for Aβ_x-40_ or Aβ_x-42_ concentration using methods previously described (Fisher et al., 2016). A mouse monoclonal anti-Aβ_40_ capture antibody (mHJ2) or anti-Aβ_42_ capture antibody (mHJ7.4) made in-house was used in conjunction with a biotinylated central domain detection antibody (mHJ5.1) and streptavidin-poly-HRP-40 (Fitzgerald Industries, Acton, MA). Super Slow ELISA TMB (Sigma-Aldrich) was then used to develop, and absorbance was read by a BioTek Epoch plate reader at 650 nm. The same assay can be used for both human and murine Aβ_x-40_. Standard curves for ELISAs were generated using synthetic human Aβ_40_ or Aβ_42_ (American Peptide, Sunnyvale, CA). Basal levels of ISF Aβ levels were calculated by averaging the Aβ concentrations taken every 90 min for 9 h prior to drug treatment. All Aβ levels for each mouse were then normalized by calculating percent of basal for each point. Mean ± SEM per group are shown.

### Western blotting

Guide cannula implantation and microdialysis were performed as described above using 2–4 month old APP/PS1 mice. 5 μM AMPA or vehicle was administered to APP/PS1 mice via reverse microdialysis for 8 or 14 h. Immediately following treatment, perfusion buffer was changed to aCSF containing 0.1% Evans Blue dye for 30 min. During this period, the area of the hippocampus directly surrounding the microdialysis probe was dyed blue, approximating the area of tissue affected by reverse microdialysis drug delivery. Following the 30-min of Evans Blue administration, the mice were sacrificed and the dyed tissue surrounding the probe was microdissected and snap frozen on dry ice, generating approximately 5-7 mg of tissue per mouse. The collected hippocampal tissue was homogenized by sonication at a 10:1 volume:wet weight in 150 mM NaCl, 50 mM Tris, pH 7.4, 0.5% deoxycholic acid, 0.1% SDS, 1% Triton X-100, 2.5 mM EDTA, and protease inhibitors. Gel electrophoresis of 20 μg protein samples was performed under reducing conditions using 4–12% Bis-Tris NuPAGE gels (ThermoFisher Scientific, Waltham, MA) and then transferred to nitrocellulose membrane. Blots were probed for glial fibrillary acidic protein (GFAP; 1:500; ThermoFisher), low density lipoprotein receptor-related protein 1 (LRP1; 1:5000; Abcam, Cambridge, MA), insulin-degrading enzyme (IDE; 1μg/mL; Abcam), neprilysin (1:1000; Millipore, Billerica, MA), matrix metalloproteinase-9 (MMP-9; 1:1000; Millipore), C-terminal fragments of APP (1:1000; Sigma-Aldrich), total soluble APP (22C11; 1:5000; Millipore), soluble APP-α (poly18268; BioLegend, San Diego, CA), soluble APP-β (poly8134; 1:1000; BioLegend), β-amyloid 1–16 (6E10; 1:500; BioLegend), glutamate receptor 2 (GluR2; 1:1000; Millipore), tubulin (1:2500; Sigma), and glyceraldehyde 3-phosphate dehydrogenase (GAPDH; 1:10,000; Sigma). HRP-conjugated goat anti-rabbit IgG (1:1000; Cell Signaling Technology, Danvers, MA) and HRP-conjugated Amersham ECL sheep anti-mouse IgG (1:1000; GE Healthcare, Chicago, IL) were used as secondary antibodies. Membranes were developed using SuperSignal West Pico Substrate (ThermoFisher) or Lumigen-TMA6 (GE Healthcare) and imaged using the Kodak ImageStation 440CF (Rochester, NY). Band intensity was quantified using the Kodak 1D Image Analysis software, and normalized using tubulin or GAPDH signals as loading controls. Values shown are these normalized band intensities relative to the experimental control group. Mean ± SEM per group are shown.

### Quantitative real-time PCR (qPCR)

Using the same tissue preparation as used for Western blotting (described above), APP/PS1 mice were treated with 5 μM AMPA for 8 or 14 h, followed by 30 min of 0.1% Evans Blue solution via reverse microdialysis. Dyed tissue around the probe was microdissected and frozen. Quantitative PCR was performed as described previously (Fisher et al., 2016). The RNeasy Mini Kit (Qiagen, Valencia, CA) was used to extract RNA, which was then reverse transcribed with a High Capacity cDNA Reverse Transcription kit (ThermoFisher). The Harvard Medical School Primer Bank was used to design primers [26–28]. Real-time detection of PCR product was performed using the Fast SYBR Green Master Mix (Applied Biosystems, Foster City, CA) in ABI 7900HT (Applied Biosystems) with the default thermal cycling program. *cFos* was used as a positive control due to its established role as a mark of neuronal activity [29]. *Gapdh* was used as a reference gene for relative expression calculations. Relative mRNA levels were calculated using the comparative Ct method using the formula 2^-ΔΔCt^. Mean ± SEM per group are shown.

### Histology

2–4 month-old wild-type mice (*n* = 6 per group) or APP/PS1 mice (*n* = 3 per group) were treated with 8 h or 14 h, respectively, of AMPA or artificial CSF via reverse microdialysis then immediately transcardially perfused with ice-cold phosphate buffer saline (PBS) with 0.3% heparin. Brains were removed, fixed in 4% paraformaldehyde for 24 h at 4 °C, then placed in 30% sucrose prior to freezing and sectioning. Coronal brain sections 50 μm wide were sliced in 300 μm intervals using a freezing sliding microtome. Sections were then immunostained to visualize astrocytes or microglia using antibodies against glial fibrillary acidic protein (GFAP; 1:500, ThermoFisher) as an astrocytic marker or against ionized calcium-binding adaptor molecule 1 (Iba1; 1:500; Wako Laboratory Chemicals, Richmond, VA) as a microglial marker. Biotinylated secondary antibody, horseradish peroxidase-conjugated streptavidin, and DAB reaction (Sigma) were used to develop. Brain sections were imaged with a Nanozoomer slide scanner (Hamamatsu Photonics, Bridgewater, NJ). Staining density was qualitatively evaluated by blinded observers and vehicle- and AMPA-treated groups were compared. Images shown are representative.

### Aβ elimination half-life

Half-life of ISF Aβ was measured using methods described previously [25]. Microdialysis was performed as detailed above and basal ISF Aβ levels were collected. Reverse microdialysis was then used to treat APP/PS1 mice with either 5 μM AMPA or vehicle for 14 h, followed by co-administration with LY411575, a potent and selective γ-secretase inhibitor (Sigma-Aldrich; 5 mg/kg in corn oil, subcutaneous injection) to block Aβ production. ISF Aβ levels were measured using sandwich ELISA, and the half-life was calculated using the slope of the semi-log plot of percent change in Aβ levels versus time. The slope was calculated based only on Aβ values that were continually decreasing, excluding points at which levels plateaued. Mean ± SEM per group are shown.

### MesoScale discovery (MSD) multiplex cytokine assay

Hippocampal tissue was collected from APP/PS1 mice treated with either vehicle (*n* = 7) or AMPA (*n* = 9) for 14 h via reverse microdialysis. Only tissue directly surrounding the probe was used. Tissue was homogenized following the manufacturer protocol in 500 mM NaCl, 50 mM Tris, pH 7.4, 0.5% deoxycholic acid, 0.1% SDS, 1% Triton X-100, 2 mM EDTA, and protease inhibitors (MesoScale Discovery, Rockville, MD, USA). Samples were assayed for interleukin(IL)-1β, IL-6, and tumor necrosis factor (TNF)-α using a custom MSD Proinflammatory Panel multiplex assay using the manufacturer’s protocol. Samples were assayed duplicate. Data analysis was performed using MSD Workbench software.

### Experimental design and statistical analysis

Littermate mice were randomly assigned into treatment groups, with equal numbers of male and females. Based on power analyses for detecting changes in ISF Aβ in microdialysis experiments, we used *n* = 4–8 mice per treatment group. A full description of statistical tests and the number of mice used can be found in the figure legends. Two-tailed unpaired *t*-tests were used to compare between two groups. One-way or two-way ANOVA was used when comparing one or two independent variables, respectively, between multiple groups. The appropriate correction for multiple comparisons was used (Sidak, Tukey, or Bonferroni; refer to figure legends). Analysis of microdialysis experiments was performed by averaging the final three data points of a specific treatment period and using one-way or two-way ANOVA with an appropriate correction for multiple comparisons. Values were accepted as significant is *p* ≤ 0.05. Data in figures are presented as mean ± SEM. Prism 6.0b for Mac OS X (GraphPad, San Diego, CA) was used for all statistical analyses.

## Results

### Local administration of AMPA decreases ISF Aβ in a dose-dependent manner

Both synaptic activity and NMDA-Rs have distinct, established roles in regulating Aβ, but the involvement of AMPA-R signaling in Aβ regulation has been largely unexplored. To address this, we used in vivo microdialysis to measure the concentration of ISF Aβ in the hippocampus of mice [9, 25]. Crucially, this technique allows us to monitor changes in ISF Aβ levels over time in freely moving mice with functional glutamatergic synapses and intact neuronal networks. Through reverse microdialysis, we are also able to locally and continuously deliver small-molecule compounds, such as AMPA, into the hippocampus without needing to cross the blood-brain barrier.

**Fig. 1:**
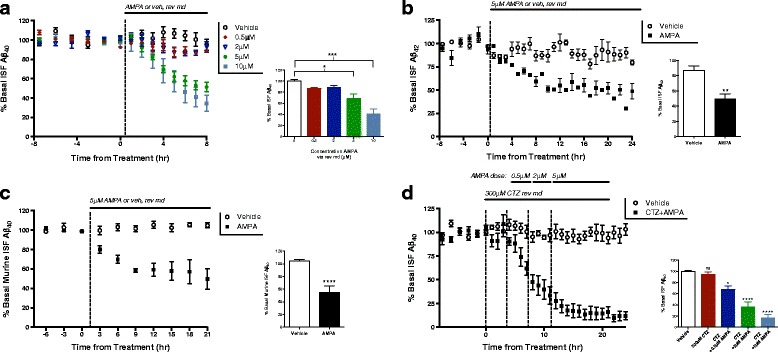
AMPA treatment decreases levels of ISF Aβ levels. **a** Varying doses of AMPA or vehicle (artificial CSF) were administered to 2–4 month-old APP/PS1 mice via reverse microdialysis (rev md), and changes in interstitial fluid (ISF) Aβ_40_ were measured using ELISA. AMPA has a dose-dependent effect on ISF Aβ levels. Though treatment with 0.5 μM and 2 μM AMPA did not alter ISF Aβ levels significantly (*n* = 3, *n* = 5 respectively), treatment with 5 μM AMPA decreased levels 31.7 ± 9.5% (*p* = 0.015, *n* = 4, one-way ANOVA, Dunnet’s post hoc test), and 10 μM AMPA decreased levels by 73.8 ± 12.2% (*p* < 0.0001, *n* = 2, one-way ANOVA, Dunnet’s post hoc test). **b** APP/PS1 mice (*n* = 4) were treated with 5 μM AMPA for 24 h and ISF Aβ_42_ levels decreased by 37.0 ± 9.4% (*p* < 0.0043, two-tailed t-test). **c** Wild-type, littermate C3H/B6 mice were dosed with 5 μM AMPA using rev md and levels of murine ISF Aβ_40_ levels decreased by 49.4 ± 8.4% (*p* < 0.0001, *n* = 6, two-tailed t-test). **d** APP/PS1 mice were treated with 300 μM cyclothiazide (CTZ) for 4 h (*n* = 6), after which increasing doses of AMPA (0.5, 2, and 5 μM) were added to the perfusion buffer. CTZ administered alone did not change ISF Aβ levels. Aβ levels decreased 31.9 ± 11.1% (*p* = 0.030, one-way ANOVA, Dunnet’s post hoc test) by 0.5 μM AMPA, 63.6 ± 11.1% (*p* < 0.0001, one-way ANOVA, Dunnet’s post hoc test) by 2 μM, and maximally decreased 83.2 ± 11.1% (*p* < 0.0001, one-way ANOVA, Dunnet’s post hoc test) when treated with 5 μM AMPA. Data plotted as mean ± SEM

Using microdialysis in the hippocampus of young, plaque-free (2–4 month old) *APPswe/PS1Δe9* hemizygous (APP/PS1) mice [23, 24], we collected hourly samples of ISF while infusing AMPA in increasing concentrations from 0.5 μM to 10 μM for 8 h each (Fig. 1a). AMPA delivered at 0.5 μM or 2 μM had no effect on ISF Aβ. However, beginning with the 5 μM AMPA concentration, ISF Aβ levels gradually decreased over time before stabilizing at a 32% decrease from baseline levels. An even greater decrease is seen following 10 μM AMPA treatment, with levels of Aβ stabilizing at a 75% decrease from baseline levels (Fig. 1a). In the following experiments, we used 5 μM AMPA in order to observe further increases and decreases in ISF Aβ levels after they are already lowered by AMPA treatment. In this study, we focus primarily on ISF Aβ_40_ because it is produced in much higher quantities than Aβ_42_ in our mouse model and therefore simpler to detect using microdialysis. To determine if AMPA treatment acts on both species of Aβ similarly, ISF samples from 5 μM AMPA-treated mice were measured for Aβ_42._ We found that AMPA decreases ISF Aβ_42_ similarly to Aβ_40_, indicating that it acts on both species of Aβ in the same manner (Fig. 1b). Next, wild-type (WT) mice were treated with 5 μM AMPA to eliminate potential confounds due to the transgenes in APP/PS1 mice. Murine ISF Aβ levels in WT animals reacted to 5 μM AMPA treatment similarly to APP/PS1 mice with a 45% decrease from baseline levels (Fig. 1c).

AMPA-Rs rapidly desensitize following AMPA or glutamate exposure [30]. One possible explanation for the observed effect on ISF Aβ, therefore, could be reduced activity due to decreased AMPA-R signaling. To test this possibility, we treated the APP/PS1 mice with cyclothiazide (CTZ), a thiazide diuretic, which inhibits desensitization and potentiates AMPA-mediated glutamate currents [31]. The mice were pre-treated with CTZ for 4 h before and then during treatment with increasing doses of AMPA (0.5 μM–5 μM) lasting 4 h each (Fig. 1d). Potentiated AMPA-R signaling enhanced the suppression in ISF Aβ levels with AMPA treatment starting at just 0.5 μM, a dose that has no effect on ISF Aβ without CTZ. This decrease is dose-dependent, with a maximal decrease in ISF Aβ of 83% from basal levels (Fig. 1d). These data indicate that the observed decrease in ISF Aβ is due to AMPA-R activity and not desensitization.

### AMPA decreases Aβ levels through multiple distinct pathways

**Fig. 2:**
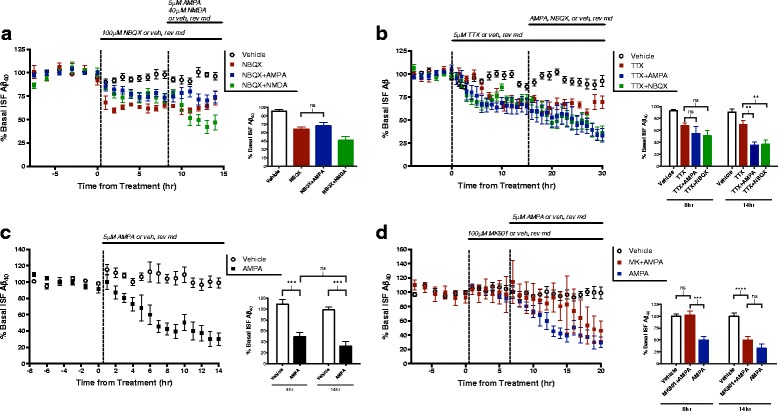
AMPA treatment alters Aβ levels through multiple pathways. **a** APP/PS1 mice (*n* = 6) were treated with 100 μM NBQX, an AMPA receptor antagonist, for 8 h then co-treated with either 40 μM NMDA (*n* = 6), 5 μM AMPA (*n* = 7), or vehicle (*n* = 12). After 6 h of co-treatment with NBQX, the addition of AMPA had no effect on Aβ levels, though NMDA still reduced Aβ by 37.5 ± 3.3% (*p* < 0.0001, one-way ANOVA, Bonferroni post hoc test). **b** Animals (*n* = 6 per group) were treated with 5 μM tetrodotoxin (TTX) for 16 h then co-treated with TTX and either 5 μM AMPA, 100 μM NBQX, or vehicle for an additional 14 h. After 8 h of co-treatment, ISF Aβ levels remained unchanged in all groups. 14 h co-treatment with AMPA reduced Aβ levels by 34.6 ± 9.9% (*p* = 0.0027, two-way ANOVA, Sidak post hoc test) and co-treatment with NBQX reduced levels by 32.8 ± 9.3% (p = 0.0027, two-way ANOVA, Sidak post hoc test). **c** APP/PS1 mice were treated with either 5 μM AMPA (*n* = 7) or vehicle (*n* = 5) for 14 h, leading to a decrease in ISF Aβ levels of 66.3 ± 11.8% (*p* = 0.0001, two-way ANOVA, Sidak post hoc test). **d** 100 μM MK801 or vehicle was administered by reverse microdialysis for 6 h to APP/PS1 mice followed by co-administration with 5 μM AMPA or vehicle. After 8 h, mice treated with AMPA alone had significance decreases in ISF Aβ as compared to vehicle-treated mice, but mice receiving both MK801 and AMPA showed no change (*p* = 0.996, two-way ANOVA, Sidak post hoc test). After 14 h, however, AMPA treatment significantly decreased ISF Aβ levels to the same extent regardless of the presence of MK801 (*p* = 0.384, two-way ANOVA, Sidak post hoc test). Data plotted as mean ± SEM

The exogenous application of AMPA through reverse microdialysis allows us to directly and selectively target AMPA-Rs. However, infusion of AMPA does not necessarily reproduce endogenous AMPA-R signaling. To address this, we treated mice with NBQX, a competitive AMPA-R antagonist (Fig. 2a). When baseline levels of AMPA-R signaling were blocked, ISF Aβ levels decreased by 32%, suggesting that AMPA-R activation increases Aβ during normal activity.

Next, we treated mice with tetrodotoxin (TTX) for 16 h to prevent the production of action potentials and therefore block evoked presynaptic release of glutamate (Fig. 2b). Following 16 h of TTX treatment, we co-infused TTX with NBQX. As previously reported, treatment with TTX alone decreases ISF Aβ levels by about 40% from basal levels [8]. Blocking AMPA-Rs in addition to TTX treatment leads to a further decrease in Aβ levels of 33% despite the cessation of presynaptic activity (Fig. 2b). Thus, AMPA-Rs activated during steady-state, tonic levels of activity appear to drive higher ISF Aβ levels independently of evoked glutamatergic signaling. Interestingly, antagonizing basally active AMPA-Rs induces a full effect on ISF Aβ levels regardless if action potentials are intact or blocked with TTX, suggesting that basal AMPA-ergic regulation of Aβ is driven by spontaneous glutamate release via miniature EPSCs (“minis”) as opposed to evoked activity.

We next determined the extent to which AMPA-mediated Aβ regulation relies on presynaptic activity. As before, mice were pre-treated with TTX followed by co-treatment with TTX and AMPA. During the initial 8 h of TTX and AMPA treatment, the decrease in Aβ levels caused by AMPA treatment (Fig. 2c) is abolished. However, a longer AMPA treatment of 14 h significantly decreased ISF Aβ levels by 30% of post-TTX levels (Fig. 2b). These results imply that, initially, evoked glutamatergic transmission is necessary for AMPA treatment to decrease ISF Aβ. With longer treatment, however, ISF Aβ levels are reduced through postsynaptic AMPA-R signaling alone, without the need of action potentials or further glutamatergic activity stimulation.

Given that high levels of NMDA-R activation result in decreased Aβ levels through calcium–dependent ERK signaling [32, 33], we hypothesized that AMPA treatment might reduce ISF Aβ levels through the indirect activation of NMDA-Rs expressed on downstream postsynaptic neurons. To determine the contribution of NMDA-Rs to the changes in Aβ levels following AMPA treatment, mice were pre-treated with MK801, a NMDA-R open channel blocker, via reverse microdialysis for 6 h before co-treatment with MK801 and 5 μM AMPA (Fig. 2d). Within the first 8 h of treatment, co-application of MK801 and AMPA does not effect an AMPA-related change in Aβ levels. The ability of AMPA to alter ISF Aβ is therefore dependent on NMDA-R activation at this time point. By hour 14 of AMPA treatment, however, Aβ levels began to decline regardless of the presence of MK801 (Fig. 2d). These data imply that AMPA’s effects on ISF Aβ levels are dependent on NMDA-R signaling for only a limited period. After prolonged treatment with AMPA, Aβ levels decrease through an NMDA-R-independent mechanism.

In consideration of these results, we questioned if AMPA-R signaling might be responsible for any part of NMDA-Rs’ effect on Aβ levels. To test this, we first treated the mice with 100 μM NBQX, a competitive AMPA-R antagonist, through reverse microdialysis then co-treated with NMDA (Fig. 2a). As observed in previous experiments [32], 40 μM NMDA reduced ISF Aβ levels to approximately 50% of basal levels within 6 h of treatment, even in the presence of an AMPA-R antagonist (Fig. 2a). Though the effect of AMPA treatment on ISF Aβ in part relies on NMDA-R involvement, the opposite does not appear true; NMDA treatment decreases Aβ levels independently from AMPA-R activation. To ensure the specificity of AMPA treatment, animals were treated with NBQX to block AMPA-Rs prior to the addition of AMPA. As was expected, NBQX completely blocked the effect of AMPA-Rs on Aβ (Fig. 2a).

### AMPA treatment results in long-lasting changes in ISF Aβ levels

**Fig. 3:**
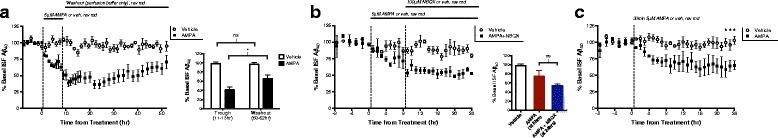
AMPA treatment results in potent, long-lasting decreases in ISF Aβ levels that slowly recover. **a** APP/PS1 mice (*n* = 5) were treated with 5 μM AMPA using reverse microdialysis for 8 h resulting in a decrease in ISF Aβ levels of 32.7 ± 3.0% from baseline. After 8 h, AMPA was removed from the microdialysis perfusion buffer. Aβ levels continued to decline for 3 h post-treatment to reach a maximum reduction of 56.7 ± 1.7% from baseline. For the next 40 h, ISF Aβ levels gradually increased. When the experiment was ended at 52 h, ISF Aβ levels had increased 23.5 ± 3.0% to reach 64.8 ± 3.0% of basal levels, which was a significant increase from the lowest Aβ levels post-treatment (*p* = 0.0245, two-way ANOVA, Sidak post hoc test). **b** APP/PS1 mice (*n* = 3) were treated with 5 μM AMPA followed by co-treatment with AMPA and 100 μM NBQX for 14 h. The addition of NBQX did not alter the decrease in Aβ levels caused by AMPA treatment (one-way ANOVA, Sidak post hoc test). **c** 5 μM AMPA was infused by rev md into APP/PS1 mice for a 30-min period, after which the perfusion buffer was changed to artificial CSF for 24 h. AMPA treatment caused a 41.30 ± 9.45% decrease in ISF Aβ levels in the 22–24 h after 30-min dosage (*n* = 3, *p* = 0.035, two-tailed t-test). Data plotted as mean ± SEM

Previous data show that activation of NMDA-R signaling rapidly decreases ISF Aβ levels by approximately 50% [32]. Once NMDA is no longer administered, ISF Aβ gradually returns to baseline levels within 30 h. AMPA treatment, however, results in a longer-lasting change in Aβ levels. APP/PS1 mice were perfused with 5 μM AMPA for 8 h. After this period, AMPA treatment ended and Aβ levels were monitored every 1–2 h for an additional 44 h (Fig. 3a). Levels of ISF Aβ decreased steadily during the AMPA treatment and continued to decrease for 3 h into the washout period to reach a maximal decrease of 60% from basal levels. From this lowest point, Aβ levels significantly increased from the trough to reach a level only 35% decreased from basal levels after 44 h of recovery (Fig. 3a). The washout study was terminated after a total of 60 h of ISF collection due to limitations in the reliable duration of microdialysis experiments, so it is possible that Aβ levels may completely recover from AMPA treatment with a longer washout period. A recovery in ISF Aβ suggests that AMPA treatment does not cause major cell death and that the area surrounding the microdialysis probe continues to function normally following treatment.

APP/PS1 mice were treated with AMPA for 8 h followed by co-administration with NBQX (Fig. 3b). The decrease in Aβ levels following AMPA application did not recover to baseline levels with the addition of NBQX despite the cessation of AMPA-R activation. Because the Aβ decrease was preserved without AMPA-ergic transmission, the effect on Aβ is likely due to a long-lasting intracellular event and not a feed-forward increase in continued glutamatergic transmission. This observed long-lived change in Aβ levels was initiated by an AMPA treatment period of only 30 min, which resulted in a 30% decrease in ISF Aβ (Fig. 3c).

### Transcription of APP processing-related genes and the levels of APP fragments are unchanged following AMPA treatment

**Fig. 4:**
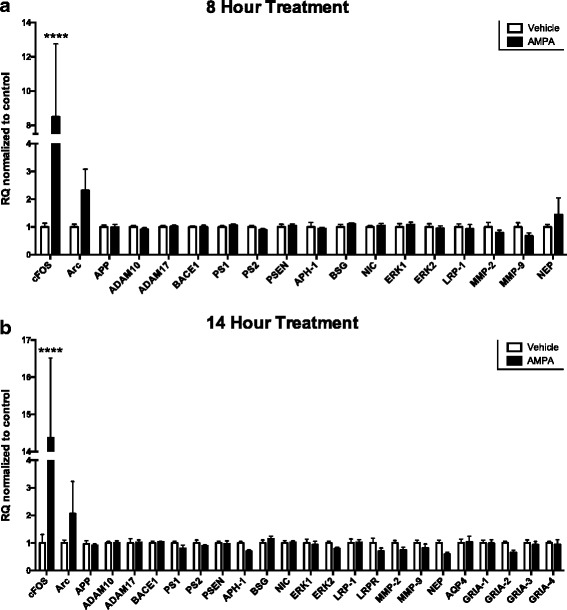
8 and 14 h AMPA treatment does not alter expression of genes related to Aβ metabolism. 5 μM AMPA or vehicle was given to 2–4 month old APP/PS1 mice for 8 h (**a**) or 14 h (**b**; *n* = 6 per group) before the hippocampal tissue surrounding the microdialysis probe was collected and analyzed with quantitative PCR. **a** qPCR analysis revealed no differences in expression for major genes involved in Aβ production and clearance between mice treated with AMPA or vehicle. Expression of *cFos*, a marker for neuronal activity, increased 7.5 ± 3.7 fold (*p* < 0.0001, two-way ANOVA, Sidak post hoc test) for the AMPA-treated group over *cFos* expression in controls. **b** After 14 h of AMPA treatment, expression of genes involved in Aβ processing was not changed as shown by qPCR analysis. AMPA-treated animals showed a 14.4 ± 1.8 fold increase in *cFos* expression over controls (*p* < 0.0001, two-way ANOVA, Sidak post hoc test). Data plotted as mean ± SEM

We demonstrated above that extended treatment with AMPA influences ISF Aβ levels without the need for NMDA-R activation. NMDA-Rs receptors are often associated with intracellular signaling and transcriptional regulation, while AMPA-Rs are generally thought of in terms of neuronal depolarization. However, there is growing evidence to suggest that AMPA-Rs may also play an active role in cellular signaling. For example, Plant et al. (2006) found that transient calcium signaling through calcium-permeable AMPA-Rs promotes the maintenance of long-term potentiation (LTP) [34]. Additionally, AMPA-R signaling, independent of depolarization, is sufficient to activate the transcription factor CREB as well as to initiate ERK signaling [17, 18, 35]. Given these results, the AMPA-R-dependent decrease in ISF Aβ that we observe could be due to the initiation of a signaling cascade by AMPA-Rs. First, we tested if AMPA-Rs affect the transcription of genes related to APP processing or Aβ clearance (Fig. 4a, b). APP/PS1 mice were administered 5 μM AMPA for 8 or 14 h by reverse microdialysis. At the end of treatment, probes were infused with Evans Blue for 30 min to mark the surrounding tissue reached by reverse microdialysis. The dyed hippocampal tissue was lysed and used for quantitative real-time PCR (qPCR) for a selection of genes involved in Aβ metabolism. Expression of the immediate early gene, *cFos*, was used as a control due to its increased expression following glutamatergic transmission [29]. As expected, AMPA treatment increased the expression of *cFos* in both the 8- and 14-h groups. However, we found no significant changes in the expression of *APP*, in genes related to α-secretase (*ADAM10* and *ADAM17*), in genes related to β-secretase (*BACE1*), nor in genes related to ϒ-secretase (*PS1, PS2, PSEN2, APH1, BSG,* and *NIC*) following 8 or 14 h of AMPA treatment (Fig. 4a, b). Further, AMPA treatment did not change expression in *ERK1* or *ERK2* or in genes associated with Aβ clearance (*LRP1, LRPR, AQP4, NEP, MMP2,* and *MMP9*). Finally, none of the AMPA-R subunits genes (*GRIA1–4*) were altered by AMPA treatment (Fig. 4a, b).

### Extended treatment with AMPA promotes increased ISF Aβ clearance

**Fig. 5:**
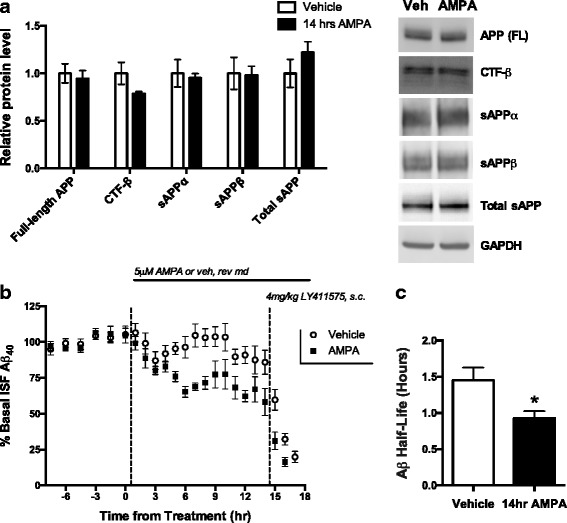
Extended treatment with AMPA decreases Aβ levels through clearance. **a** 2–4 month old APP/PS1 mice were treated with either 5 μM AMPA (*n* = 6) or aCSF (*n* = 8) via reverse microdialysis for 14 h. Tissue surrounding the microdialysis probe was analyzed via Western blot for full-length APP, CTF-β, sAPPα, sAPPβ, and total sAPP, and no significant change was observed between treatment groups (two-way ANOVA, Sidak post hoc test). Bands were normalized to GAPDH and displayed relative to control. Blot images are representative examples. **b** APP/PS1 mice were treated with 14 h of AMPA (*n* = 6) or vehicle (*n* = 7). With microdialysis collection ongoing, animals were administered a 4 mg/kg subcutaneous injection (s.c.) of LY411575, a γ-secretase inhibitor, or vehicle (corn oil). **c** ISF Aβ half-life for each treatment group was calculated by taking the slope of the semi-log plot of concentration versus time for the time points between drug delivery and the plateauing of Aβ concentrations. Mice treated with 5 μM AMPA had an Aβ half-life of 0.9 ± 0.1 h compared to a half-life of 1.5 ± 0.2 h for the mice treated with aCSF (*p* = 0.0298, two-tailed t-test)

To the best of our knowledge, all previous studies investigating the relationship between synaptic signaling and alterations in Aβ levels, including several from our laboratory, have found that synaptic signaling primarily affects Aβ production [4, 6–8, 25, 36]. However, after 14 h of AMPA administration, we found no change in full-length APP levels or in the cleavage products β-C-terminal fragment (β-CTF), soluble APP-α (sAPP-α), and sAPP-β as determined by Western blot (Fig. 5a). In combination with the lack of transcriptional changes in production-related genes (Fig. 4a, b), these data suggest that extended treatment with AMPA does not have a pronounced effect on Aβ production. It is important to note, however, that small changes in gene or protein levels, such that occur when only a subpopulation of cells is affected, can be masked when total brain lysates are analyzed. Considering the large effect that AMPA has on ISF Aβ levels, though, we hypothesized that AMPA-Rs act on ISF Aβ through a different mechanism, namely by altering its clearance.

**Fig. 6:**
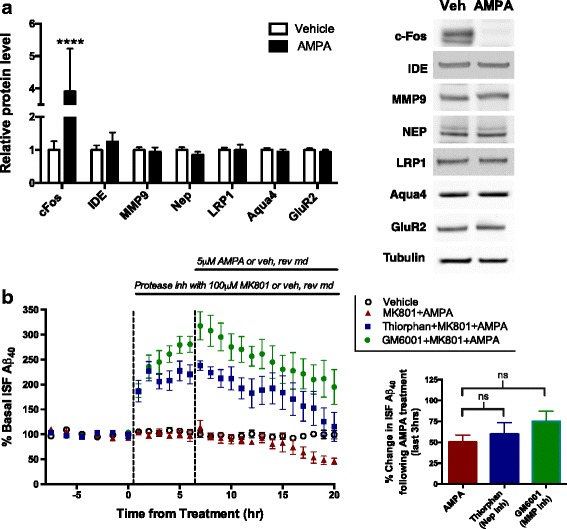
AMPA-mediated decrease in Aβ not due to changes in clearance-related proteins or proteases. **a** 2–4 month old APP/PS1 mice were treated with either 5 μM AMPA (*n* = 6) or aCSF (*n* = 8) via reverse microdialysis for 14 h. Tissue surrounding the microdialysis probe was analyzed via Western blot to determine levels of proteins involved in Aβ elimination and clearance. Bands were normalized to GAPDH and displayed relative to control. Blot images are representative examples. cFos protein expression was increased 2.9 ± 0.4 fold (*p* < 0.0001, two-way ANOVA, Sidak post hoc test) in the AMPA group compared to the controls. No other proteins showed a significant difference between treatment groups. **b** Reverse microdialysis was used to treat APP/PS1 mice (*n* = 7) with 10 μM thiorphan (neprilysin inhibitor), 25 μM GM6001 (broad-spectrum MMP inhibitor), or vehicle for 6 h, followed by 14 h of co-treatment with 5 μM AMPA. The Aβ concentrations in the last 3 h of each treatment were averaged and the differences between the end of inhibitor/vehicle treatment and after the addition of AMPA were compared. Inhibiting protease activity with thiorphan or GM6001 did not alter the decrease in ISF Aβ levels observed following AMPA treatment (*p* = 0.40, one-way ANOVA, Dunnet’s post hoc test). Data plotted as mean ± SEM

Aβ is eliminated from the ISF through five main pathways: receptor-mediated transport across the blood brain barrier (BBB), enzymatic degradation, cellular uptake, glymphatic-mediated clearance, or passive bulk-flow clearance for (reviews see [37–39]). If any of these pathways is targeted by AMPA treatment, the rate of ISF Aβ clearance could increase. To test this possibility, we measured half-life of ISF Aβ in mice treated with either 5 μM AMPA or vehicle using reverse microdialysis (Fig. 5b). AMPA treatment leads to a rapid decrease in Aβ that stabilizes by 6–8 h of treatment. After 14 h, mice were subcutaneously injected with LY411575, a potent γ-secretase inhibitor that rapidly inhibits Aβ production. LY411575 enters the brain and within 15 min reaches a concentration approximately 200-fold in excess of its IC_50_ for γ-secretase inhibition [25]. Once γ-secretase is inhibited, all new production of Aβ is precluded and microdialysis is used to monitor the levels of remaining ISF Aβ over time. The rate at which Aβ in the ISF is eliminated can be measured by calculating the slope of the semi-log plot of percentage baseline Aβ levels versus time. This elimination rate was determined for both groups, and the Aβ half-life calculated. Interestingly, the half-life of ISF Aβ was significantly shorter by over 30% in mice receiving AMPA treatment (t_1/2_ = 0.93 h) than those in the control group (t_1/2_ = 1.38 h), indicating that AMPA treatment increases the clearance of ISF Aβ (Fig. 5c). It is important to note that 6 of 12 AMPA-treated mice had ISF Aβ levels decrease so much that a reliable half-life could not be calculated. If this greater decrease following AMPA treatment was also due to enhanced clearance, then the observed effect of AMPA on Aβ would be even greater so, we could be underestimating the effect of AMPA on Aβ clearance. Next, we measured the levels of key proteins involved in Aβ clearance in the hippocampal tissue surrounding the microdialysis probe for mice treated with 14 h of AMPA or vehicle (Fig. 6a). Similar to the qPCR experiments (Fig. 4b), only the positive control cFos showed a significant change in protein levels with AMPA treatment (Fig. 6a). Though these data suggest that none of the Aβ clearance-related proteins selected is involved in AMPA-mediated regulation of Aβ, Western blots do not detect cell type-specific changes in protein levels, alterations in protein function, or changes in protein localization. To test if AMPA treatment increases protease activity and thus Aβ degradation, we pre-treated APP/PS1 mice with the neprilysin inhibitor, thiorphan, or with the broad-spectrum metalloproteinase (MMP) inhibitor, GM6001, before co-treating with AMPA. Inhibition of neprilysin and all MMP family members both blocks Aβ clearance pathways and potentially inhibits α-secretase, which increases ISF Aβ levels when those agents are administered singly (Fig. 6b). Importantly, the addition of AMPA still decreased Aβ by a comparable amount as observed without the protease inhibitors, indicating that AMPA does not affect degradation of Aβ through these proteases.

### AMPA-R activation does not induce broad inflammation

**Fig. 7:**
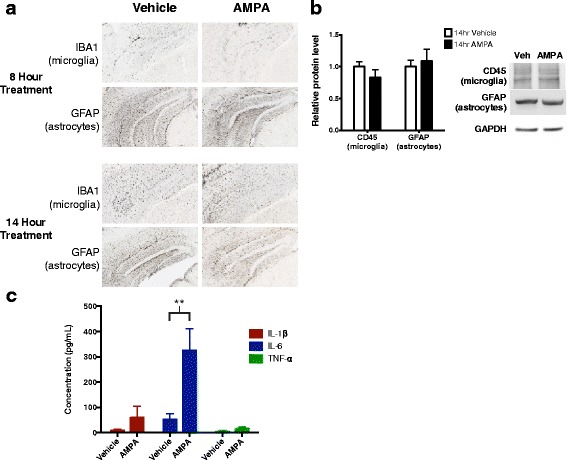
Glial recruitment unchanged and IL-6 levels enhanced following AMPA treatment. **a** Wild-type C3H/B6 mice (for the 8 h treatment, *n* = 6 per group) or APP/PS1 mice (for the 14 h treatment, *n* = 3 per group) were implanted with microdialysis probes and treated with either 5 μM AMPA or aCSF for 8 or 14 h. Brain sections were immunostained with DAB using anti-GFAP antibody to mark astrocytes or anti-Iba1 antibody to mark microglia. Immunoreactivity between control and AMPA-treated sections were compared, and representative images are shown. **b** 2–4 month old APP/PS1 mice were treated with either 5 μM AMPA (*n* = 6) or aCSF (*n* = 8) via reverse microdialysis for 14 h. Tissue surrounding the microdialysis probe was analyzed via Western blot for GFAP or CD45, markers of astrocytes and microglia, respectively, and no difference was observed between treatment groups (two-way ANOVA, Sidak post hoc test). Bands were normalized to GAPDH and displayed relative to control. Blot images are representative examples. **c** As in Fig. 7b, APP/PS1 mice were treated with either 5 μM AMPA (*n* = 9) or vehicle (*n* = 7) for 14 h, and hippocampal lysates were analyzed for pro-inflammatory cytokines using a MSD multiplex assay. Levels of IL-1β (*p* = 0.991, two-way ANOVA, Sidak post hoc test) and TNF-α (*p* = 0.999, two-way ANOVA, Sidak post hoc test) were unchanged. IL-6 levels were significantly elevated following AMPA treatment, increasing from 52.3 to 773.8 pg/mL (*p* = 0.0014, two-way ANOVA, Sidak post hoc test). Data plotted as mean ± SEM

A potential concern is that AMPA treatment decreases ISF Aβ by causing cellular toxicity and/or creating a lesion through increased glutamatergic activity [40]. If AMPA does cause cellular damage, an inflammatory response would involve the recruitment and activation of microglia and astrocytes [41–43]. To monitor inflammatory responses, mice were treated with 5 μM AMPA or with vehicle for 8 or 14 h before brains were collected and fixed in 4% formaldehyde. The brains were stained for Iba1, a marker of microglia [44], and GFAP, a marker for astrocytes [42, 45]. As expected, we found increased Iba1 and GFAP staining around the microdialysis probe tract, but no change in staining density between the AMPA- and vehicle-treated tissue at either time point (Fig. 7a). For confirmation, we measured protein levels of GFAP and CD45, another microglial marker [46], using hippocampal lysates from APP/PS1 mice treated with either 5 μM AMPA or vehicle for 14 h (Fig. 7b). In agreement with the immunostaining results, AMPA treatment did not increase GFAP or CD45 protein levels, indicating a lack of glial recruitment (Fig. 7b). In addition to monitoring the glial response, we measured pro-inflammatory cytokines levels in the hippocampal lysates of mice following AMPA treatment. Though IL-1β and TNF-α levels were unchanged, the levels of IL-6 showed a dramatic increase of over 500% (Fig. 7c). IL-6 is a neuropoietic cytokine with both neuromodulatory and neuroprotective roles, known to be induced by neuronal activity [47–49]. Without a visible increase in gliosis and with no significant increase in IL-1β or TNF-α, there does not appear to be a broad inflammatory response. These data, along with the partial recovery of ISF Aβ in the 44 h sampled following AMPA treatment (Fig. 3a), strongly suggest AMPA is not causing widespread toxicity accounting for the effects on Aβ observed in this study.

**Fig. 8:**
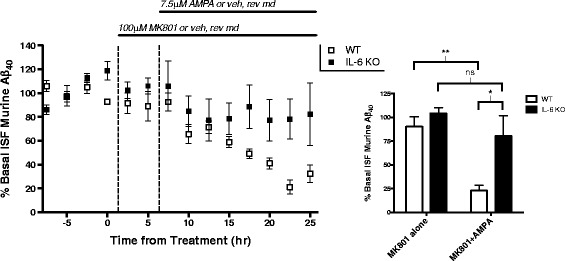
IL-6 is required for AMPA-R regulation of ISF Aβ levels. Both IL-6^−/−^ mice (*n* = 5) and C3H/B6 WT mice (*n* = 6) were treated with 100 μM MK801 for 6 h via reverse microdialysis, then co-treated with MK801 and 7.5 μM AMPA for an additional 17 h. The last five hours of each treatment (MK801 alone vs MK801 + AMPA) were averaged for each treatment group and compared (two-way ANOVA, Sidak post hoc test). In WT animals, ISF Aβ levels decreased by 67.34% from MK801 alone to MK801 + AMPA (*p* = 0.002). In IL-6^−/−^ animals, the addition of AMPA resulted in a non-significant decrease in ISF Aβ levels of 23.96% (*p* = 0.652). Furthermore, ISF Aβ levels IL-6^−/−^ mice following extended AMPA treatment are significantly higher than observed in WT mice (80.26 and 23.0%, respectively; *p* = 0.027). Data plotted as mean ± SEM

IL-6 is a neuropoietic cytokine with both neuromodulatory and neuroprotective roles, known to be induced by neuronal activity [47–49]. Intriguingly, IL-6 has previously been linked to enhanced Aβ clearance [50, 51]. Because levels of IL-6 increased greatly following AMPA treatment, we tested the possibility that enhanced IL-6 signaling is involved in the decrease in ISF Aβ levels following AMPA-R stimulation. To do this, we utilized 3-month-old IL-6-deficient mice (IL-6^−/−^ mice). These mice develop normally and produce normal levels of murine Aβ in the ISF. We treated IL-6^−/−^ and WT mice with MK801 to block NMDA-R signaling for 6 h, then added 7.5 μM AMPA into the perfusion buffer for an extended period (Fig. 8). Because murine Aβ levels are much lower in these mice than in our amyloidosis models, samples were collected every 2.5 h. Similar to our observations in APP/PS1 mice, AMPA treatment led to a decrease in ISF Aβ by approximately 67% in WT mice. Conversely, AMPA failed to produce a significant change in ISF Aβ levels in IL-6^−/−^ mice, suggesting that IL-6 signaling is necessary for AMPA-R regulation of Aβ.

## Discussion

**Fig. 9:**
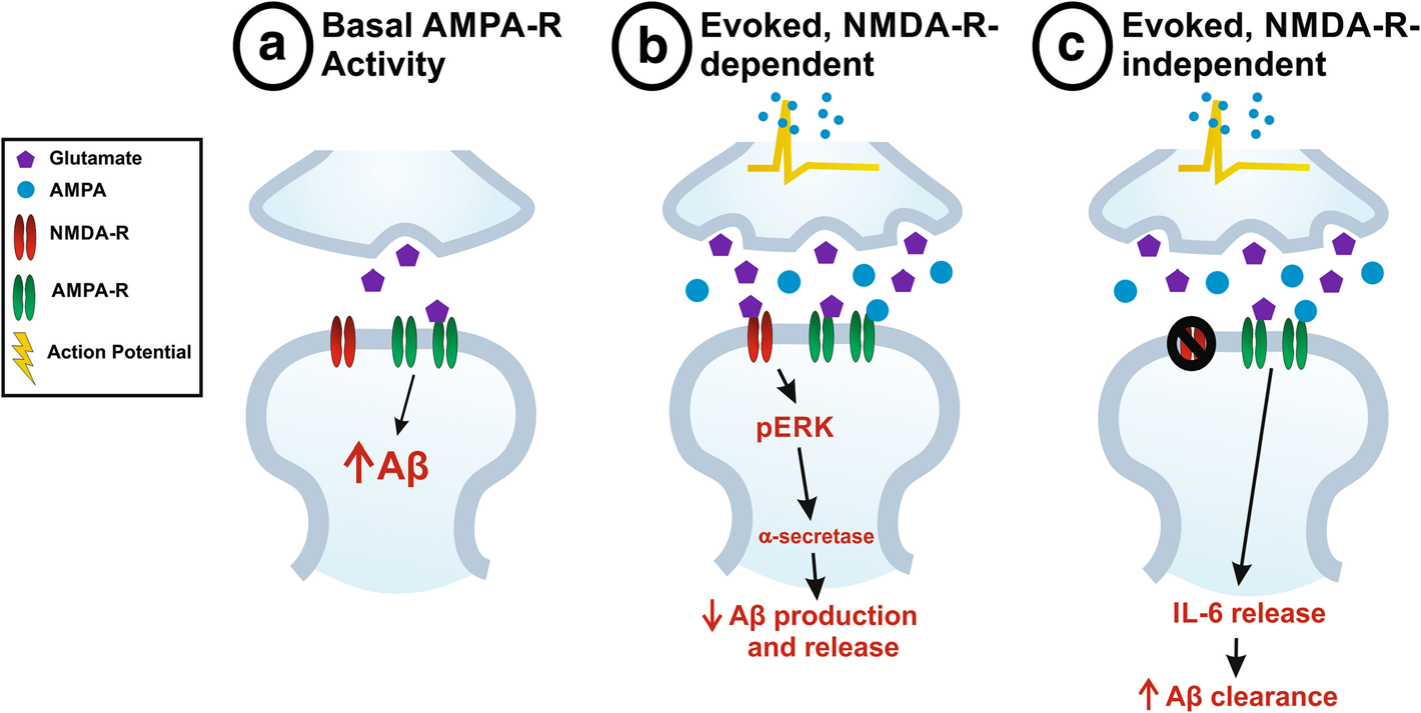
Model of AMPA-R-mediated Aβ regulation. **a** Tonic, steady-state AMPA-R activity driven by spontaneous neurotransmission increases levels of Aβ in the ISF. **b** Evoked glutamatergic transmission resulting from AMPA treatment initially decreases ISF Aβ through NMDA-R activation. As described in previous studies, NMDA-Rs lead to decreased Aβ production and release into the ISF through ERK phosphorylation and enhanced α-secretase activity. **c** Extended AMPA-R activation, independent of NMDA-Rs, increases IL-6 signaling to stimulate clearance of Aβ from the ISF

In this study, we provide evidence that though steady-state levels of AMPA-Rs encourage heightened ISF Aβ levels, evoked AMPA-R signaling decreases extracellular Aβ concentration through two different pathways (see Fig. 9 for model). The first of these pathways acts on Aβ through an indirect network effect; AMPA-R stimulation increases glutamatergic transmission, including elevated NMDA-R signaling on the postsynaptic neuron. It has been previously shown that NMDA-Rs regulate Aβ levels by using calcium as a second messenger to activate ERK and increase α-secretase activity. Second, we found that AMPA-Rs can also influence Aβ levels independently of NMDA-Rs. This purely AMPA-R-mediated pathway takes longer to recruit, increases the rate of ISF Aβ clearance, and requires IL-6 signaling. Gene expression and protein levels of many primary clearance-related molecules remain unchanged, possibly indicating cell-type specific changes or alterations in protein function or localization.

### Exogenous application of AMPA decreases ISF Aβ through postsynaptic signaling

We found that infusion of AMPA directly into the hippocampus of APP/PS1 mice through reverse microdialysis decreases ISF Aβ levels by up to 75% following the maximal dose of 10 μM. Treatment with AMPA induces a potent, long-lasting effect on Aβ levels, with even a brief application initiating a full response. AMPA-Rs, therefore, appear to be significant regulators of Aβ levels in the extracellular space. Factors that influence extracellular levels of Aβ have the potential to directly influence AD pathogenesis by altering the likelihood of Aβ to aggregate [52]. That AMPA increases activity but suppresses Aβ levels is somewhat surprising considering previous reports that synaptic activity drives production of Aβ. Treatment with the GABA_A_ receptor antagonist picrotoxin, high levels of potassium chloride, or electrical stimulation promotes Aβ secretion into the extracellular space [7–9]. In a more physiological setting, increasing activity within the barrel cortex through vibrissal stimulation results in higher levels of ISF Aβ in APP/PS1 mice [4, 53]. In humans, the highest levels of amyloid deposition are found in brain regions with the highest baseline metabolic activity [10].

Considering these findings, it would be reasonable to hypothesize that AMPA-Rs, as excitatory channels, should increase Aβ levels. Paradoxically, however, we found increasing AMPA-R activation through exogenous AMPA treatment significantly decreases ISF Aβ. Because AMPA-Rs are susceptible to rapid desensitization, we considered the possibility that AMPA-Rs act on Aβ levels through induced synaptic depression [30, 31]. However, when receptor desensitization was blocked with cyclothiazide, the decrease in Aβ in response to AMPA was potentiated. Receptor desensitization only limited Aβ suppression, and receptor activation is directly responsible for the reduction of Aβ levels.

Though general increases in synaptic activity upregulate Aβ production, the activation of certain postsynaptic signaling systems can alter APP processing to yield varied effects on Aβ levels, particularly when α-secretase is targeted. As mentioned above, serotonin receptor activation decreases Aβ levels through PKA and ERK activation [12, 13]. The serotonin receptor illustrates the specificity involved in Aβ regulation; only the G_s_-linked receptors decrease Aβ whereas the other G-protein coupled serotonin receptors have no effect or increase Aβ [13]. Additionally, M1 muscarinic acetylcholine (mACh) receptor agonists decrease Aβ production, and deleting this receptor leads to increased Aβ and amyloid pathology [54–56]. Within the glutamate receptor family, muscarinic glutamate receptor 5 has been shown to trigger Aβ production [57, 58], and NMDA-Rs can modulate Aβ levels bidirectionally [32, 33, 59]. Clearly, postsynaptic effects on Aβ are diverse and markedly context-specific.

### Spontaneous and evoked AMPA-R activation differentially regulate Aβ levels

In these studies we have shown that AMPA-R regulation of Aβ is multifarious (see model Fig. 9). When basal AMPA-R activity is antagonized, ISF Aβ decreases by 20%. The same decrease occurs even after action potentials are blocked and evoked synaptic transmission is inhibited, indicating that the basal AMPA-R signaling that increases Aβ levels is likely due to spontaneous transmission (Fig. 9a). Conversely, application of AMPA via reverse microdialysis causes direct AMPA-R activation as well as stimulates evoked glutamatergic transmission. In this scenario, AMPA-R activation decreases Aβ levels. This dual effect of AMPA-Rs, depending on the mode of transmission, has been seen in various contexts. Sara and colleagues (2011) utilized a use-dependent AMPA-R antagonist to show that spontaneous and evoked transmission activate discrete populations of AMPA-Rs [60]. Additionally, several studies found that receptors that respond differentially to spontaneous and evoked transmission are physically and functionally distinct [61–66]. Intriguingly, spontaneous activity appears to suppress protein synthesis while evoked activity stimulates translation. Another possible explanation is that the effects of AMPA-Rs on Aβ are dependent on relative levels of AMPA-R activation. During basal transmission, a smaller set of AMPA-Rs is active compared to the AMPA-Rs targeted by action potentials or exogenous AMPA treatment. How endogenous AMPA-Rs promote increased levels of ISF Aβ remains unknown, though we speculate that basal AMPA-ergic signaling induces amyloidogenic APP processing through increased endocytosis within or near the presynaptic terminal, as described in previous studies [7–9, 67].

### Extended AMPA treatment decreases ISF Aβ half-life

Adding an additional layer of complexity, exogenous AMPA treatment appears to act on Aβ levels through two distinct pathways. Within the first 8 h of treatment, AMPA’s ability to modulate Aβ levels depends on NMDA-R signaling (Fig. 9b). This pathway relies on presynaptic activity to increase glutamatergic transmission, thus stimulating NMDA-R activation on downstream neurons to decrease Aβ production in these cells [32, 33]. The reverse is not true, however; AMPA-Rs do not appear to play a role in NMDA-R-mediated decreases in Aβ. Following longer AMPA treatment, a novel pathway by which AMPA-Rs influence Aβ independently of both presynaptic activity and NMDA-Rs emerges.

As detailed above, studies regarding synaptic and postsynaptic regulation of Aβ have primarily addressed the effects of activity on Aβ production. However, we were unable to detect changes in APP processing-related gene expression or in APP fragment levels in hippocampal lysates following either 8 or 14 h of AMPA treatment. Instead, using microdialysis along with a potent γ-secretase inhibitor, we found that treatment with AMPA for 14 h decreased the half-life of ISF Aβ, implying that AMPA-Rs modulate Aβ clearance (Fig. 9c). This does not appear to involve glial recruitment, a broad inflammatory response, or changes in key clearance-related proteins. We found that one proinflammatory cytokine, IL-6, increased dramatically following AMPA treatment. IL-6 has been shown to have both normal physiological as well as inflammatory, pathological roles in the CNS [49, 51, 68, 69] and has been shown to be secreted in response to neuronal depolarization [47, 48]. Furthermore, IL-6 signaling has been linked to increased Aβ clearance through microglial phagocytosis [50, 51]. Given the substantial increase in IL-6 following AMPA treatment, we propose that AMPA could be causing IL-6 release and enhanced phagocytosis of Aβ. In support of this hypothesis, mice deficient in IL-6 fail to show decreased ISF Aβ levels in response to AMPA treatment, suggesting that IL-6 signaling is involved in AMPA-R regulation of ISF Aβ levels. The IL-6 receptor is expressed on neurons, microglia, and astrocytes [70, 71], so this synaptic activity-dependent clearance pathway could be mediated by multiple cell types. Though we propose a connection between neuronal IL-6 release and microglial clearance, our data do not indicate which cell types are involved. Furthermore, we have only tested a handful of cytokines in response to AMPA treatment thus far, leaving open the possibility that multiple cytokines are involved in this pathway. Future experiments will address the mechanism through which AMPA-Rs affect Aβ clearance.

The finding that AMPA treatment decreases Aβ levels is supported by a previous study by Hoey and colleagues conducted in primary cortical neurons [22]. Unlike our study, however, the authors conclude that Aβ production is decreased when AMPA directly acts to increase non-amyloidogenic APP processing. In contrast, our in vivo studies suggest that AMPA treatment requires an intermediary step of NMDA-R activation in order to increase non-amyloidogenic processing of APP. Additionally, our studies model a second pathway in which AMPA directly acts on Aβ through enhanced clearance. Because this pathway likely involves multiple cell types interacting, experiments using neuronal cultures would not recapitulate the effects we observed. Furthermore, the discrepancies in findings could also be explained by developmental differences between our two systems. Hoey et al. (2013) found that AMPA-mediated alterations in APP processing are at least partially due to calcium-permeable AMPA-Rs. There is evidence that GluA2, the receptor subunit responsible for determining the receptor’s calcium permeability, is developmentally regulated [72–75]. Finally, we have found that even slight changes in AMPA concentration can change Aβ’s response, and our two studies used very different doses. We administered 5 μM AMPA through the microdialysis probe of which only an estimated 10% diffuses into the extracellular space. In contrast, Hoey et al. (2013) administered 50 μM AMPA, potentially activating a different pathway than we observed.

Though both production and clearance determine the steady state levels of Aβ in the extracellular space, late-onset AD (LOAD) is primarily characterized by dysfunctions in Aβ clearance [38, 76]. In 2003, we found that ISF Aβ half-life as measured by microdialysis is doubled in an aged APP transgenic model compared to young animals [25]. In human studies, metabolic labeling and CNS analysis revealed impaired clearance rates in participants with LOAD, though Aβ production was unaltered [76]. Furthermore, many of the genetic factors associated with LOAD are related to clearance, including *APOE*, *CLU*, *CR1*, and *CD33*. Given the evident prominence of Aβ clearance in AD, our results highlight the importance of understanding the ways in which synaptic activity impinges on previous clearance-related studies.

## Conclusions

There are clearly numerous mechanisms that together regulate Aβ levels. Though the confluence of these various synaptic-mediated pathways appears to result in increased Aβ, we propose that certain postsynaptic signaling pathways, such as those described in these studies, act as protective mechanisms that aid in maintaining Aβ homeostasis. The failure of these Aβ-suppressing pathways may contribute to the breakdown of homeostasis that ultimately results in the build-up of pathology. Indeed, glutamatergic transmission is one of the first systems targeted by toxic species of amyloid as the disease progresses [77–80].

As the dominant excitatory ionotropic receptors in the brain, AMPA-Rs have the potential to greatly influence extracellular Aβ levels and amyloid pathology. We have found that activation of AMPA-Rs initiates a varied and complex response in which opposing pathways act concurrently to regulate Aβ levels. Our results link postsynaptic signaling through AMPA-Rs to the increased release of IL-6 and enhanced Aβ clearance. Soluble, monomeric Aβ production is a normal process of every brain. Even those brains destined to develop AD pathology produce Aβ for decades without formation of toxic aggregates. The point at which Aβ becomes pathogenic is likely influenced by a number of factors, including the loss of homeostatic pathways. Identifying and understanding how, early in our lives, Aβ levels are controlled may give us clues to disease etiology or even prevention.

## Abbreviations

aCSF: Artificial cerebrospinal fluid
AD: Alzheimer’s disease
AMPA-R: AMPA receptors
APP: Amyloid precursor protein
APP/PS1: *APPswe/PS1ΔE9*
Aβ: Amyloid-β
BBB: Blood brain barrier
BSA: Bovine serum albumin
CTZ: Cyclothiazide
ERK: Extracellular regulated kinase
GAPDH: Glyceraldehyde 3-phosphate dehydrogenase
GFAP: Glial fibrillary acidic protein
Iba1: Ionized calcium-binding adaptor molecule 1
IDE: Insulin-degrading enzyme
IL: Interleukin
ISF: Interstitial fluid
LOAD: Late-onset AD
LRP1: Low density lipoprotein receptor-related protein 1
LTP: Long-term potentiation
mACh: Muscarinic acetylcholine
MMP: Matrix metalloproteinase
NMDA-R: NMDA receptor
qPCR: Quantitative real-time PCR
TNF: Tumor necrosis factor
TTX: Tetrodotoxin
WT: Wild-type
β-CTF: β-C-terminal fragment
